# Alloimmune Activation Promotes Anti-Cancer Cytotoxicity after Rat Liver Transplantation

**DOI:** 10.1371/journal.pone.0091515

**Published:** 2014-03-20

**Authors:** Stéphanie Lacotte, Graziano Oldani, Florence Slits, Lorenzo A. Orci, Laura Rubbia-Brandt, Philippe Morel, Gilles Mentha, Christian Toso

**Affiliations:** 1 Department of Surgery, Geneva University Hospitals, University of Geneva, Geneva, Switzerland; 2 Hepato-pancreato-biliary Centre, Geneva University Hospitals, University of Geneva, Geneva, Switzerland; 3 Department of Pathology, Geneva University Hospitals, University of Geneva, Geneva, Switzerland; Centre de Recherche Public de la Santé (CRP-Santé), Luxembourg

## Abstract

Liver transplantation for hepatocellular carcinoma (HCC) results in a specific condition where the immune response is potentially directed against both allogeneic and cancer antigens. We have investigated the level of anti-cancer immunity during allogeneic immune response. Dark Agouti-to-Lewis and Lewis-to-Lewis rat liver transplantations were performed and the recipients anti-cancer immunity was analysed at the time of alloimmune activation. The occurrence of rejection in the allogeneic recipients was confirmed by a shorter survival (*p*<0.01), increased liver function tests (*p*<0.01), the presence of signs of rejection on histology, and a donor-specific *ex vivo* mixed lymphocyte reaction. At the time of alloimmune activation, blood mononuclear cells of the allogeneic group demonstrated increased anti-cancer cytotoxicity (*p*<0.005), which was related to an increased natural killer (NK) cell frequency (*p*<0.05) and a higher monocyte/macrophage activation level (*p*<0.01). Similarly, liver NK cell anti-cancer cytotoxicity (*p*<0.005), and liver monocyte/macrophage activation levels (*p*<0.01) were also increased. The alloimmune-associated cytotoxicity was mediated through the NKG2D receptor, whose expression was increased in the rejected graft (*p*<0.05) and on NK cells and monocyte/macrophages. NKG2D ligands were expressed on rat HCC cells, and its inhibition prevented the alloimmune-associated cytotoxicity. Although waiting for *in vivo* validation, alloimmune-associated cytotoxicity after rat liver transplantation appears to be linked to increased frequencies and levels of activation of NK cells and monocyte/macrophages, and is at least in part mediated through the NKG2D receptor.

## Introduction

Liver transplantation is the most effective treatment for patients with early unresectable hepatocellular carcinoma (HCC) [1], [2]. However, 15% of recipients experience post-transplant HCC recurrence, which quickly leads to death in almost all patients [3]. Various strategies have been proposed to decrease this risk, including an improved transplant selection criteria, the targeting of circulating HCC cells, the use of adjuvant anti-cancer drugs and a promoted anti-cancer immunity [4].

Transplantation for HCC is a unique condition, with immune activation directed against both allogeneic donor and cancer antigens. This dual activation has rarely been explored. An alloimmune activation may only be directed against specific allogeneic antigens or be linked to a broader activation also promoting a non-specific anti-cancer immune response. The latter hypothesis has been suggested by a number of studies showing a higher risk of post-transplant HCC recurrence in patients with more profound immune inhibition; for example, after the use of anti-lymphocyte antibodies or in case of overexposure to calcineurin inhibitors [5]–[8]. In addition, a decreased expression of one NKG2D ligand on HCC tumors, low neutrophil-lymphocyte blood ratios and tumor-associated macrophage counts have also been associated with HCC recurrence [9]–[12].

Ideally, the allogeneic immunity should be prevented and the anti-cancer immunity promoted. A better understanding of the cross-talk between the two is therefore desirable, in order to better define the mediators and the mechanisms involved in each type of immunity. Ultimately, such data will help define the ideal immunosuppression combination after liver transplantation for HCC.

The present study assesses the level of anti-cancer cytotoxicity in the liver, spleen and blood after allogeneic rat liver transplantation. It defines the role of specific immune cell types, including natural killer (NK) cells and monocyte/macrophages, and the action of key NK cell receptors.

## Material and Methods

### Animals, Liver Transplantation and Ethics Statement

Experiments were performed on male Lewis and Dark Agouti (DA) rats weighing 200–250 g (7 to 8 weeks-old, Janvier). They underwent orthotopic liver transplantation according to a protocol previously described [13]. DA-to-Lewis transplantations (allogeneic model) were considered as the study group, and Lewis-to-Lewis transplantations were used as syngeneic controls (six animals in each group for the survival assessment). All animals were cared for according to the international guidelines on Animal Care, and ethical approval was obtained from the ethical committee at the University of Geneva and from the Geneva veterinary authorities (N°1052/3653/3).

### Rat HCC cell lines

JM-1 cells were kindly provided by George Michalopoulos (University of Pittsburg) [14]. McA-RH7777 cells were purchased from ATCC (Molsheim, France). Both cell lines were cultured in DMEM medium at high glucose level (Gibco).

### Liver Function Test Assessment, Liver Histology and Immunolabelling

To detect signs of liver rejection, serum liver function tests, including aspartate aminotransferase (AST), alanine aminotransferase (ALT) and bilirubin were assessed on day one, three and ten after transplantation. Serum levels were measured in collaboration with the central clinical hospital laboratory (Synchron LX20). Samples from nine animals in each group were analysed.

The presence of rejection was also assessed on histology after hematoxin/eosin staining of liver samples retrieved on day ten after transplantation. The level of rejection was blindly graded by an expert liver pathologist according to the Banff classification [15]. NK cells were labeled on cryosections with anti-NKRP1 (CD161, 10/78, Biolegend) followed by Alexa Fluor®555 donkey anti-mouse IgG (Invitrogen). Macrophages were labeled on paraffin-embedded sections with polyclonal rabbit anti-Iba1 (Wako) followed by Alexa Fluor®555 donkey anti-rabbit IgG (Invitrogen).

### Peripheral Blood, Spleen and Liver Mononuclear Cell Isolation

Tail vein blood samples (500 μl) were collected in 150 μl of acid citrate dextrose. Liver and spleen were retrieved through a midline abdominal incision after 10 U IV heparin injection. The liver was perfused with HBSS containing 0.5 mg/ml of collagenase D (Sigma). It was cut in small pieces, resuspended in HBSS/collagenase solution, digested at 37°C for 20 min. Cell suspension was washed and filtered through a nylon mesh. The spleen was cut in pieces, crushed and filtered through a nylon mesh. Peripheral blood, liver and spleen cells were purified in a Ficoll Paque (GE Healthcare) density gradient. Isolated mononuclear cells were washed and counted.

### Mixed Lymphocyte Reaction and ELISA

Peripheral and spleen allogeneic immune activations were tested using mixed lymphocyte reactions. Cell incubations were performed in 96 well-plates in 200 µL RPMI 1640 (Gibco), 10% FCS (Invitrogen), 1 M HEPES (Invitrogen) and 1 U/ml/1 μg/ml Penicillin-Streptomycin and 0.29 mg/ml L-glutamine (Gibco). Responder Lewis cells (2.5×10^5^) from four allogeneic and four syngeneic recipients were incubated with 2.5×10^5^ irradiated stimulator DA (or third part) splenocytes (5000 rad) at 37°C/5% CO_2_ for 24 hours.

The level of interferon gamma (IFNγ) was quantified by ELISA. Polystyrene plates (Costar) were coated overnight at 4°C with purified anti-rat γIFN (1∶500, clone DB-1, Biolegend) in carbonate buffer. Plates were washed and saturated for 30 min with supplemented RPMI 1640 medium. Cell culture supernatants were added for two hours, followed by biotin anti-rat IFNγ (1∶1000, Poly5109, Biolegend), streptavidin-horseradish peroxidase (1∶1000, Biolegend) and the substrate solution (TMB reagent, R&Dsystem). The level of IFNγ was obtained according to a dilution curve.

### Flow Cytometry Antibodies and Cytotoxicity Assay

The phenotype of blood, liver and spleen mononuclear immune cells was assessed by flow cytometry. Labeling was performed using antibodies against CD3 (1F4) (BD Pharmingen), CD4 (W3/25) (Biolegend), CD8 (OX-8), CD172a (OX-41) for monocyte/macrophages and NKRP1 (CD161, 10/78) for NK cells. The cell phenotype was assessed in nine animals per group.

NK cell receptors were assessed using anti-NKG2D (11D5F4) (eBiosciences) and anti-NKp30 (sc-33647) (SantaCruz) antibodies. The presence of NKG2D-ligands was tested on JM-1 and McA-RH7777 cell lines after incubation with mouse recombinant NKG2D-Fc chimera (139-NK-050, R&D Systems)(5 μg/well) and anti-human IgG Fc (HP6017, Biolegend).

The level of anti-cancer cell cytotoxicity was assessed with peripheral blood mononuclear cells (PBMC) and NK cells from the liver (six animals per group), the spleen and the blood (seven animals per group). They were incubated with CFSE-labeled Yac-1 target cancer cells (0.5 μM, Invitrogen) at decreasing effector/target ratios ranging from 40/1 to 4/1 for four hours at 37°C. Of note, Yac-1 cells lack the major histocompatibility complex class I and are commonly used for cytotoxic assays. Following labeling with AlexaFluor®647-AnnexinV (1.5 μl, Biolegend) and 7-AAD (1/1000, Invitrogen) in annexin binding buffer, dead cells were assessed using FACS Calibur (BD).

In selected experiments, the inhibition of cytotoxicity was tested upon incubation with anti-NKG2D and anti-NKp30 antibodies (1 μg/well).

### NK Cell Magnetic Sorting

In order to specifically look at NK cells, a negative sorting was performed from PBMC and splenocytes. After incubation with mouse anti-CD172a (OX-41, GeneTex), anti-CD45RA (OX-33, Biolegend) and anti-TCR (R73, Biolegend) antibodies, cells were washed with a buffer (PBS 2% BSA 2 mM EDTA), and Dynabeads® coated goat anti-mouse IgG (Invitrogen) were added. After washing, the cell suspension was applied on a magnet in order to remove non-NK cells. The NK cell purity was assessed by flow cytometry (85% in the spleen and 90% in the blood).

Liver NK cells were obtained by a two-step negative/positive selection. Liver mononuclear cells were incubated with biotinylated anti-CD172a and anti-biotin antibodies coupled to microbeads (MiltenyiBiotec). After removal of the CD172a+ cells through a column, the flow-through was incubated with anti-CD161 FITC and anti-FITC microbeads allowing the positive selection of NK cells. The NK cell purity was assessed by flow cytometry (80% in the liver). Sorted cells were immediately used for phenotyping or cytotoxic assay.

### Real-Time Polymerase Chain Reaction (qPCR)

Total RNA (cells or liver biopsies) was prepared and purified using the RNeasy Mini Kit (Qiagen, Germantown, MD) according to the manufacturer's instructions. One μg of cDNA was synthesized by extending a mix of random primers with the High Capacity cDNA Reverse Transcription Kit in the presence of RNase Inhibitor (Applied Biosystems). The relative quantity of each transcript was normalized according to the expression of rplp1 (ribosomal protein large P1). Primer sequences for *rplp1*, *nkg2d*, *rrlt*, *rae1l* and *irp94* were designed with AmplifX and Primers3 softwares and are available upon request. Amplification reactions were performed in a total volume of 20 µl using a Thermocycler sequence detector (BioRad CFX96) with qPCR Core kit for SYBR Green I (Eurogentec).

### Statistical analysis

Results were represented as median +/− IQR. Results were statistically compared using Mann-Whitney test. Survivals were plotted on Kaplan-Meier curves and compared with the log-rank test. Statistical significance was set at p<0.05.

## Results

### Assessment of Alloimmunity and Rejection

**Figure 1:**
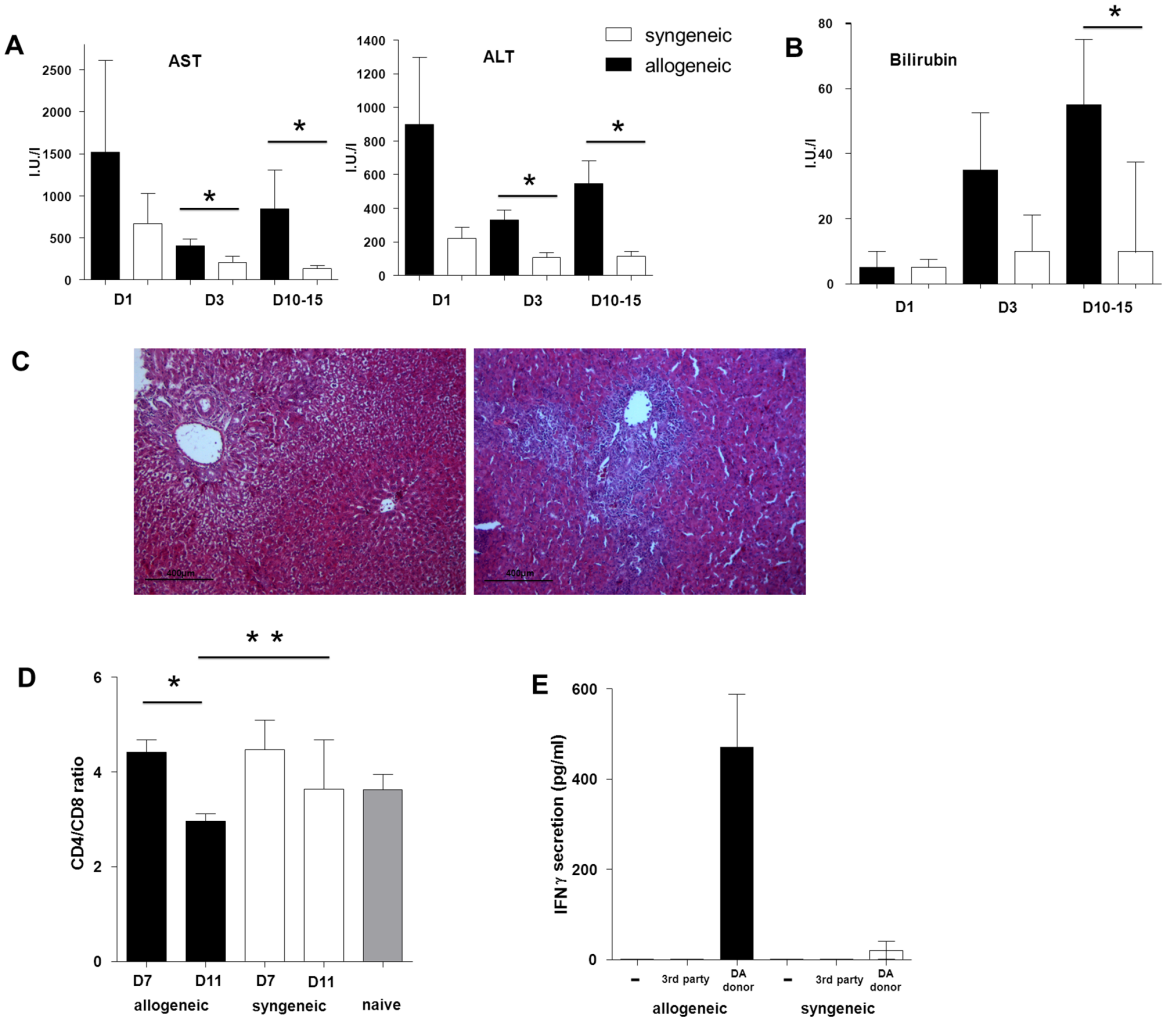
Assessment of alloimmunity and rejection. (A) Serum aspartate aminotransferase (AST) and alanine aminotransferase (ALT) levels after allogeneic Dark Agouti-to-Lewis (black) and syngeneic Lewis-to-Lewis (white) rat liver transplantations. (B) Bilirubine levels after allogeneic (black) and syngeneic (white) transplantation. (C) Representative hematoxilin-eosin stained biopsies on day 10 after allogeneic (right) and syngeneic (left) rat liver transplantation. (D) Post-transplant blood CD4/CD8 ratios on day 7 (D7) and 11 (D11). (E) IFNγ secretion after peripheral blood mononuclear cells (PBMC) mixed lymphocyte reaction on day ten post-transplantation with donor (DA) and third part stimulation. *p<0.05, **<0.01.

The presence of an alloreactivity was specifically looked at because some allogeneic rat-to-rat liver transplantation models induce only low levels of alloimmunity without acute cell rejection [16]. As described in the literature, the studied DA-to-Lewis orthotopic rat liver transplantations were linked to shorter survivals compared to the syngeneic transplantations (median survival: 13 vs. >60 days, p = 0.0037, data not shown). Serum liver function tests (AST and ALT) were increased after day three in the allogeneic recipients (all p<0.01 vs. syngeneic controls, Fig. 1A). Bilirubin level was increased after 10 days in the allogeneic recipients (p = 0.026 vs. syngeneic controls, Fig. 1B).

The presence of cell rejection was confirmed on the day 10 histology and was graded Banff 8 in all studied allogeneic recipients. Syngeneic control recipients were graded Banff 0 (Fig. 1C). In addition, the peripheral CD4/CD8 ratio on day 11 was significantly lower in the allogeneic recipients compared to syngeneic recipients (2.9 *vs*. 3.6, p = 0.0051, Fig. 1D). Finally, the alloimmunity was specifically directed towards donor antigens as demonstrated by the day 10 mixed lymphocyte reactions against DA splenocytes (supernatant IFNγ concentration: 410 pg/ml *vs*. undetectable), and the absence of response against third party Fisher rat antigens (Fig. 1E).

### Alloimmunity-Associated Peripheral Cytotoxicity

**Figure 2:**
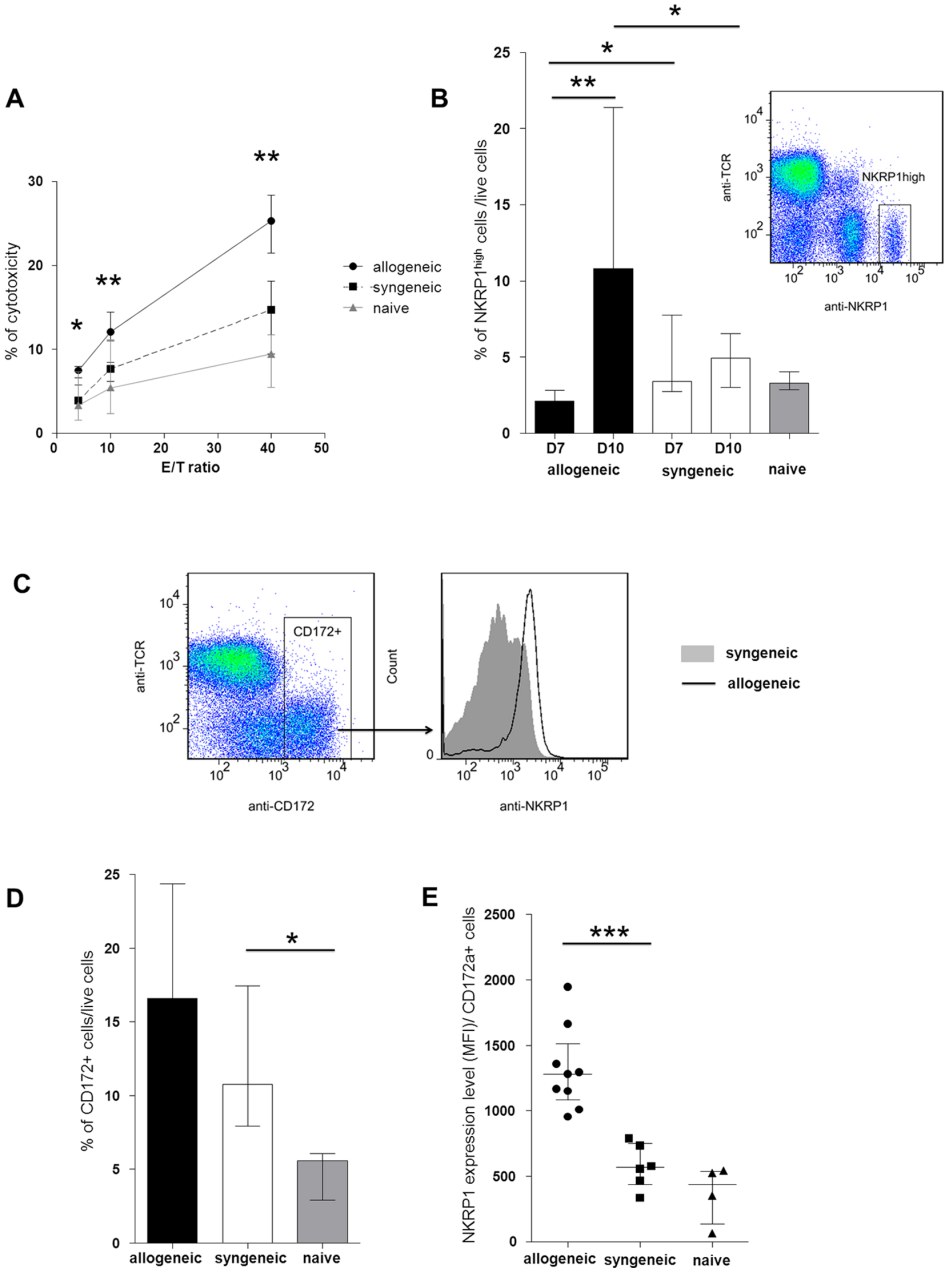
Alloimmunity-associated peripheral cytotoxicity. (A) *Ex vivo* PBMC anti-cancer cytotoxicity on day ten after allogeneic and syngeneic transplantations and in naïve control rats at various effector/target ratio (E/T). (B) Frequency of NKRP1^high^ cells (NK cells) among PBMCs on day 7 (D7) and 10 (D10) after transplantation. Flow cytometry dot-plot assessed the gating strategy. (C) Flow cytometry dot-plot and histogram assessing the gating strategy for monocytes/macrophages (CD172+) and NKRP1 expression level on CD172+ cells. (D) Frequency of monocyte/macrophage (CD172+ cells) among PBMCs. (E) Activation level of monocyte/macrophages (NKRP1 expression level (mean fluorescence intensity (MFI)) among CD172a+ cells). *p<0.05, **<0.01.

In an effort to assess the impact of rejection on anti-cancer immunity, the cytotoxic activity of peripheral immune cells was tested *ex vivo* against cancer Yac-1 cells. At the time of alloimmune activation (day 10), the level of peripheral cytotoxicity was increased in the allogeneic recipients compared to the syngeneic controls and naïve rats (effector/target 40/1: 25.2% *vs*. 14.7% and 9.4% respectively, p = 0.0025, Fig. 2A). This pattern was observed at all tested dilutions (4/1, 10/1, 40/1).

In order to assess whether the altered cytotoxicity was related to a variation in the cell subsets, flow cytometry analyses were conducted on PBMCs. NKRP1 (CD161), a C-type lectin membrane glycoprotein, is highly expressed on NK cells and was used as a marker for rat NK cells [17]. On day seven, the NK cell frequency (CD3^−^ NKRP1^high^) was significantly decreased in the allogeneic recipients compared to the syngeneic controls (2.1% *vs*. 3.4%, p = 0.028, Fig. 2B). This decreased frequency of peripheral NK cells is in line with the previously observed migration of NK cells into the liver early after allogeneic transplantation [18]. On day 10, the observed increased peripheral cytotoxicity was related to an increased blood NK cell frequency (10.79% in allogeneic *vs*. 4.9% in syngeneic, p = 0.048).

Monocytes/macrophages can also be involved in the cytotoxic events against cancer cells. The day 10 monocyte population (CD172a^+^) was increased in both allogeneic and syngeneic recipients compared to the naïve non-transplanted controls, likely reflecting some degree of non-specific peri-operative immune activation (16.6% *vs*. 10.7% *vs*. 5.58%, respectively; p = 0.1 for allogeneic *vs*. naïve; p = 0.023 for syngeneic *vs*. naïve, Fig. 2D). This said, the highest frequency was observed in the allogeneic recipients (yet not reaching statistical significance, p = 0.455 for allogeneic *vs*. syngeneic). In addition, the frequency of activated monocytes expressing NKRP1 (CD161) was higher in the allogeneic recipients compared to the syngeneic controls and the naïve non-transplanted animals (mean fluorescence intensity 1282 *vs*. 568 *vs*. 438, p = 0.0004, Fig. 2E) [19].

### Absence of Alloimmunity-Associated Spleen Cytotoxicity

**Figure 3:**
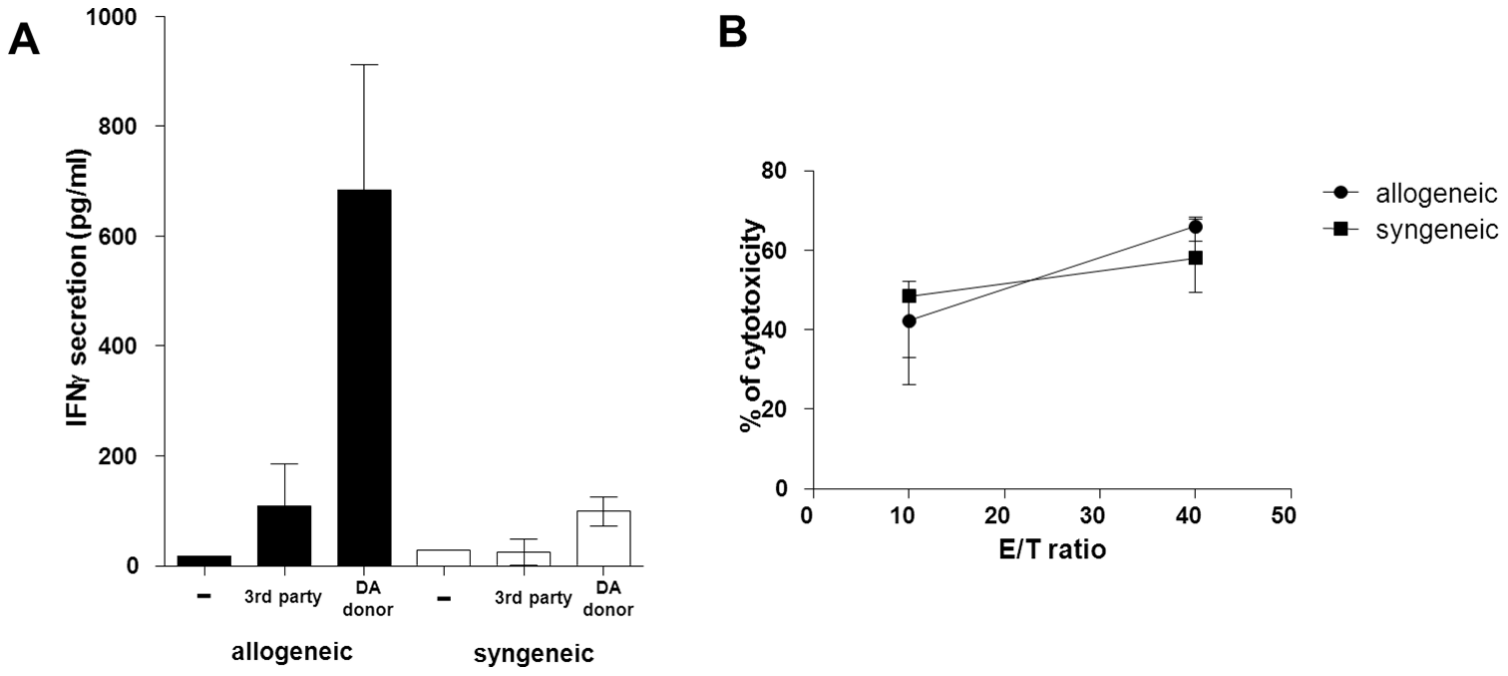
Alloimmunity-associated spleen cytotoxicity on ten days after transplantation. (A) IFNγ secretion after splenocyte mixed lymphocyte reaction on day ten post-transplantation with donor and third part stimulation. (B) *Ex vivo* spleen NK cell anti-cancer cytotoxicity on day ten after allogeneic and syngeneic transplantation.

In order to better characterise the observed peripheral cytotoxicity, we assessed the allogeneic and anti-cancer immune events in the spleen. The day 10 splenocytes from allogeneic rats stimulated with donor cells (DA) secreted more IFNγ than the splenocytes from syngeneic rats, confirming the presence of a donor-specific allogeneic response in the spleen (684 *vs*. 99 pg/ml, p = 0.028, Fig. 3A). Conversely, sorted spleen NK cells demonstrated no difference of cytotoxicity between allogeneic and syngeneic recipients (effector/target ratio 10/1: 42.2 vs. 48.3%, p = 0.30, Fig. 3B). Similarly, no alteration in the number and the phenotype of spleen cells could be detected (data not shown).

### Alloimmunity-Associated Liver Mononuclear Cell Activation

The immune response of liver mononuclear cells was tested on day 10 after transplantation. After isolation, the number of liver mononuclear cells was increased in the allogeneic rats compared to their syngeneic counterparts (16×10^6^ *vs*. 5.35×10^6^ cells/liver, p = 0.004).

**Figure 4:**
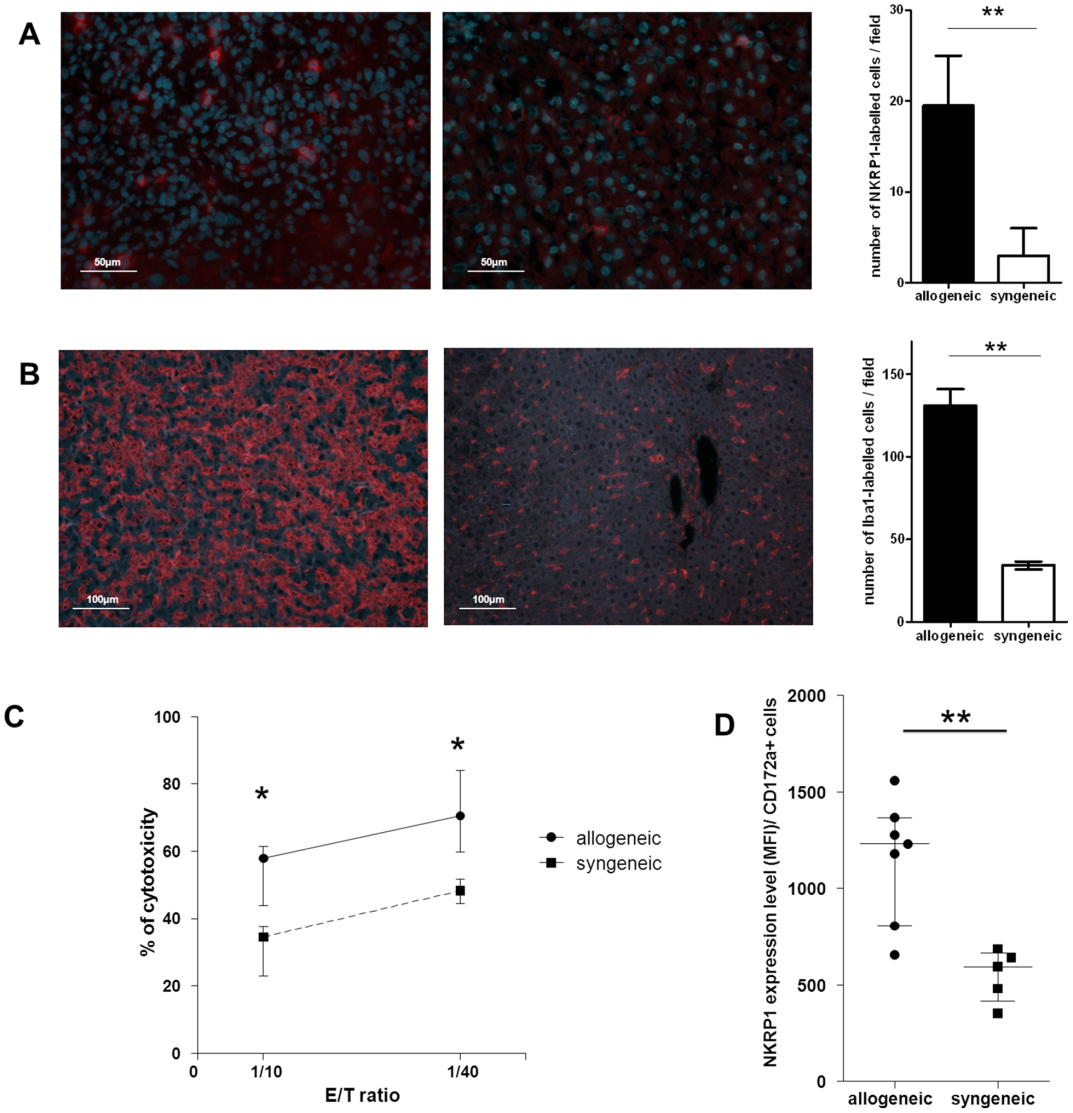
Alloimmunity-associated liver mononuclear cells cytotoxicity on ten days after transplantation. (A) Representative images of sections of allogeneic (left) and syngeneic (right) liver graft labeled with anti-NKRP1 antibodies (red). Nuclei were stained with Hoechst (blue). Number of labeled-NK cells per image counted on multiple liver sections. (B) Representative images of sections of allogeneic (left) and syngeneic (right) liver graft labeled with anti-Iba1 antibodies (red). Number of labeled macrophages per image counted on multiple liver sections. (C) *Ex vivo* liver NK cell anti-cancer cytotoxicity on day ten after allogeneic and syngeneic transplantation. Two effector/target ratio (E/T) were tested. (D) Activation level of liver monocyte/macrophages (NKRP1 expression level (MFI) among CD172a+ cells). *p<0.05, **<0.01.

This rise of the mononuclear cells number was related to an increased number of NK cells in the allogeneic graft (19.5 *vs*. 3 in the syngeneic graft, p = 0.006, Fig. 4A) and to an increased number of macrophages (131 *vs*. 34.5 in the syngeneic graft, p = 0.0034, Fig. 4B). These two cells subsets were found both in the hepatic parenchyma and in cell infiltrates of the allogeneic graft. The observed alteration of cell number was associated to an increased activation. Sorted liver NK cells demonstrated an increased anti-cancer cytotoxicity in the allogeneic recipients (effector/target ratio 10/1: 58.8% *vs*. 32%, p = 0.030, Fig. 4C). Of note, allogeneic rat liver transplant recipients showed a trend towards a higher cytotoxicity in liver-sorted NK compared to spleen-sorted NK cells (effector/target ratio 10/1: 58.8% vs 42.2%, p = 0.101). This higher liver NK cell activity is likely related to the allogeneic rejection primarily located in the liver graft [20].

The level of liver monocyte activation was increased in the allogeneic recipients (MFI: 1232 *vs*. 590, p = 0.005, Fig. 4D).

### Alloimmunity-Associated Cytotoxicity through the NKG2D receptor

**Figure 5:**
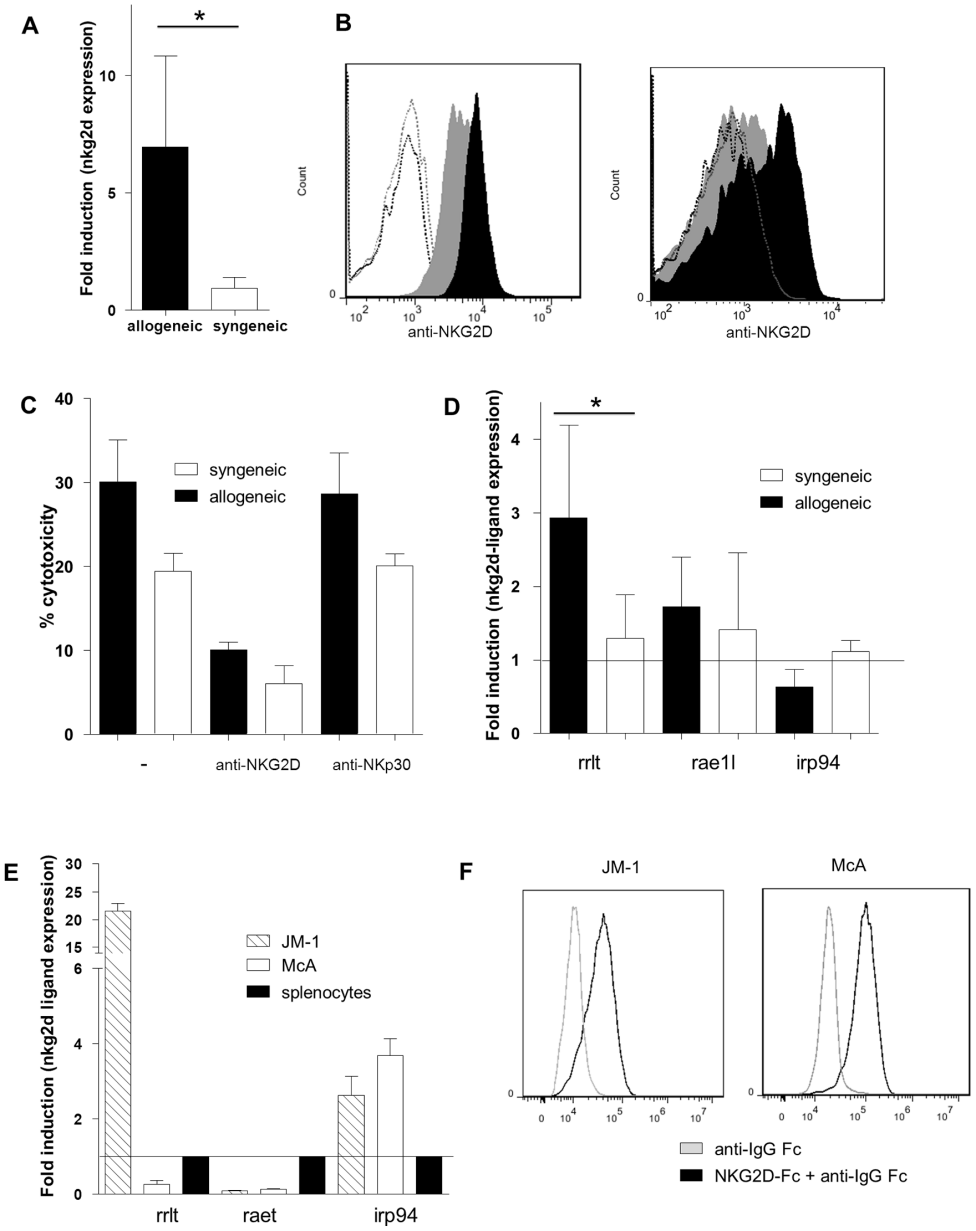
Alloimmunity-associated cytotoxicity is mediated through the NKG2D receptor. (A) Liver expression of *nkg2d* on day ten after liver transplantation. (B) Representative NKG2D expression levels in blood NK cells (left) and monocytes (right) of allogeneic (black) and syngeneic (grey) recipients. Isotype was used as control (dashed lines). (C) Sorted blood NK cell cytotoxicity inhibition with anti-NKG2D antibody or with anti-NKp30 antibody. (D) Levels of NKG2D ligand (*rae1l, rrlt* and *irp94*) expression in the liver on day ten after transplantation. (E) Levels of NKG2D ligand (*rae1l*, *rrlt* and *irp94*) expression in rat HCC cell lines. (F) Representative level of recombinant NKG2D-Fc binding to rat HCC cells lines. *p<0.05, **<0.01.

In order to define potential receptor/ligand pathways involved in the detected cytotoxicity, qPCR were performed on day nine liver biopsies. Similar to a previous report, the expression of *nkg2d*, one of the best characterised tumor-related NK receptor, was 6.9 fold higher in the allogeneic liver grafts compared to the syngeneic controls (p = 0.028, Fig. 5A) [21]. As NKG2D receptor is constitutively expressed on NK cells, this result could have been related to the higher number of NK cells in the allogeneic liver. However, the levels of expression of NKG2D in the blood NK cells (median MFI: 7694 vs. 4636, Fig. 5B, left) and monocytes (median MFI: 1180 vs. 666, Fig. 4B, right) were also higher in allogeneic recipients.

The implication of the NKG2D receptor in cytotoxicity was tested with the use of anti-NKG2D antibody. Sorted blood NK cells demonstrated higher cytotoxic levels in the allogeneic animals, and this effect was prevented by the addition of anti-NKG2D antibody (30.1 *vs*. 10.1, Fig. 5C). This inhibition was receptor-specific as no decrease could be observed with the use of anti-NKp30 antibody.

The expression of NKG2D ligands was further assessed by qPCR on day nine liver biopsies. In rat, three NKG2D ligands were described: Rae1l, Rrlt (retinoic acid early transcript family) and Irp94 (ischemia responsive 94 kDa protein, heat shock protein family) [21], [22]. The *rrlt* gene expression was significantly increased in the allogeneic liver grafts compared to syngeneic liver grafts (2.93 fold increase, p = 0.028, Fig. 5D).

In an effort to determine how the increased NKG2D-mediated cytotoxicity contributed to HCC cell clearance, the expression of NKG2D ligands was also assessed on two rat HCC cell lines, JM-1 and McA. *Rrlt* was expressed in JM-1 cell line (21.4 fold increase compared to primary splenocytes), but not in McA cells. *Rae1l* was not expressed in HCC cells and *irp94* was expressed at different levels in the two cell lines (JM-1: 2.63, McA: 3.68 fold increase, Fig. 5E). Finally the presence of NKG2D ligands at the surface of HCC cell lines was confirmed with the use of a chimeric recombinant NKG2D-Fc. Both JM-1 and McA expressed high levels of known and potentially still unknown NKG2D ligands (MFI, JM1: 3.53.10^4^ *vs*. 1.02.10^4^, McA: 9.4.10^4^ *vs* 1.83.10^4^, Fig. 5F).

Overall, NKG2D-ligands were expressed in all HCC cell lines supporting the idea that activated NKG2D-expressing NK cells and monocytes contribute to HCC cell clearance.

## Discussion

This study provides novel insights to the question of the balanced immune activation after allogeneic liver transplantation in the presence of HCC, by demonstrating the presence of an alloimmune-dependent anti-cancer cytotoxic activity after rat liver transplantation. This cytotoxicity is linked to increased frequencies and levels of activation of NK cells and monocyte/macrophages, and is at least in part mediated through the NKG2D receptor.

The studied Dark Agouti-to-Lewis rat liver transplantation model induced a strong alloimmune activation specifically directed against donor antigens [23]. This immune profile was associated with an increased phenotype and level of activation of NK cells and monocytes/macrophages. This is in accordance with previous studies demonstrating the harmful impact of macrophages during a rat kidney allograft rejection and the protective effect of NK cell depletion after allogeneic rat liver transplantation [18], [24].

The cross-talk between the allogeneic and anti-cancer immunities has been poorly studied so far, and the present data show an enhanced liver and peripheral (but not spleen) anti-cancer cytotoxicity at the time of liver graft rejection. In human organ transplantation, NK cells were present in endomyocardial cell infiltrates but these cells d, but o not appear as major players in liver graft rejection [25], [26]. In fact, an increased alloreactivity of NK cells after liver transplantation has been reported and this activity was not correlated with rejection episodes [26]. However, human liver and secondary lymphoid tissue NK cells have shown strong levels of cytotoxicity after stimulation by IL-2 or in the presence of activated APCs [27], [28].

While waiting for *in vivo* and/or clinical data, the present observation supports the use of milder levels of immunosuppression in patients transplanted for HCC, in order to preserve the anti-cancer cytotoxicity. Of note, many allogeneic rat-to-rat liver transplantation models induce no acute cell rejection, and the perfect model with syngeneic –recipient type- HCC cells and acute allogeneic immunity is currently missing (no Lewis HCC cell line available), thus preventing the *in vivo* validation of the presented data. Also, the described anti-cancer activity was observed after advanced rejection events, which are seldom seen clinically. However, we hypothesize that milder levels of rejection also promote cancer cell clearance.

Rather than decreasing immunosuppression as a whole, a careful drug selection may also be attempted to spare NK cells and monocyte/macrophages in view of a better HCC cell clearance. The use of depleting anti-lymphocyte antibody has been associated to an increased risk of post-transplant HCC recurrence [8]. Non-depleting anti-IL2-R antibodies may also have an effect, as they alter the phenotype and function of newly produced NK cells [29]–[31]. Sirolimus and mycophenolate mofetil have been shown to alter the NK cell phenotype and function *in vitro*, while cyclosporine does not appear to have an effect [32]. *In vivo*, all maintenance drugs have an impact on various immune cell subsets and the ideal immunosuppression combination remains to be defined in the setting of liver transplantation for HCC [33], [34].

The NKG2D receptor/NKG2D ligands pathway appeared as a key player in the alloimmune-associated cytotoxicity. It was already published that NKG2D expression level is raised in liver allogeneic graft [21]. Here, we have demonstrated that the expression of NKG2D is specifically increased in peripheral NK cells and macrophages at the time of rejection, and the *ex vivo* cytotoxicity of liver NK cells is prevented by blocking the NKG2D receptor (but not the NKp30 receptor). In addition, NKG2D ligands could be found on rat HCC cells, confirming human data with the expression of UL16-binding protein (ULBP) 1, a human NKG2D ligand, on HCC tumors [10]. This pathway has been previously explored in other oncological settings such as colorectal carcinoma, and the level of tumor NKG2D ligand expression has been associated to HCC response and patient survival [10], [35]. NKG2D expression on blood NK cells is increased after radiofrequency HCC ablation, and is linked to higher disease-free survival rates [36]. This rise in NKG2D expression is also observed after transarterial chemoembolization and is correlated with a decrease in NKG2D-ligand shedding (soluble MICA) [37]. NKG2D expression after liver transplantation might help in HCC cells clearance. Finally, NKG2D has been recently involved in the macrophage-NK cell cross-talk leading to higher levels of NK cell activation and anti-tumor cell cytotoxicity [38]. The observed increased expression of NKG2D on macrophages at the time of allogeneic liver graft rejection may contribute indirectly to cytotoxicity by promoting NK cell activation and cytotoxicity.

Beyond NKG2D, a wide panel of activating and inhibitory receptors is expressed on NK cells, which pattern of expression can modulate the level of activation of NK cells [39]. To illustrate, inhibitory receptors can recognize class I major histocompatibility complex, and inhibit NK cell cytotoxicity [40]. The receptor profile differs between organ-specific NK cells, as do their function [41], and changes in the balance between activating and inhibitory receptors could explain at least some of the herein described phenotypic and functional NK cell differences between sites in syngeneic and allogeneic recipients. Further exploration is deserved.

The present data demonstrate that the occurrence of a rat liver allograft rejection promotes anti-cancer cytotoxicity through the NKG2D receptor. While waiting for *in vivo* validation, the observed *ex vivo* cytotoxicity argue in favor of the use of milder and tailored immunosuppression protocols in patients undergoing liver transplantation for HCC.
